# Organic Farming Favours Insect-Pollinated over Non-Insect Pollinated Forbs in Meadows and Wheat Fields

**DOI:** 10.1371/journal.pone.0054818

**Published:** 2013-01-28

**Authors:** Péter Batáry, Laura Sutcliffe, Carsten F. Dormann, Teja Tscharntke

**Affiliations:** 1 Agroecology, Georg-August University, Göttingen, Germany; 2 MTA-ELTE-MTM Ecology Research Group, Budapest, Hungary; 3 Plant Ecology and Ecosystem Research, Georg-August University, Göttingen, Germany; 4 Computational Landscape Ecology, UFZ Centre for Environmental Research, Leipzig, Germany; 5 Biometry and Environmental System Analysis, University of Freiburg, Freiburg, Germany; Cirad, France

## Abstract

The aim of this study was to determine the relative effects of landscape-scale management intensity, local management intensity and edge effect on diversity patterns of insect-pollinated vs. non-insect pollinated forbs in meadows and wheat fields. Nine landscapes were selected differing in percent intensively used agricultural area (IAA), each with a pair of organic and conventional winter wheat fields and a pair of organic and conventional meadows. Within fields, forbs were surveyed at the edge and in the interior. Both diversity and cover of forbs were positively affected by organic management in meadows and wheat fields. This effect, however, differed significantly between pollination types for species richness in both agroecosystem types (i.e. wheat fields and meadows) and for cover in meadows. Thus, we show for the first time in a comprehensive analysis that insect-pollinated plants benefit more from organic management than non-insect pollinated plants regardless of agroecosystem type and landscape complexity. These benefits were more pronounced in meadows than wheat fields. Finally, the community composition of insect-pollinated and non-insect-pollinated forbs differed considerably between management types. In summary, our findings in both agroecosystem types indicate that organic management generally supports a higher species richness and cover of insect-pollinated plants, which is likely to be favourable for the density and diversity of bees and other pollinators.

## Introduction

Agricultural intensification is a major driver of biodiversity loss, affecting not only endangered species but increasingly also common and generalist species [1], [2]. Agricultural intensification can occur on different spatial scales, from local and landscape to regional scales [3], [4], [5]. Common types of local-scale intensification are the increased use of agrochemicals and deep ploughing in arable crops, and increased grazing and mowing in grasslands. During the last two decades the importance of landscape-scale intensification has also been recognised, including landscape simplification due to the loss of non-crop natural habitats such as hedges and natural field boundaries.

In order to counteract the negative effects of agricultural intensification, agri-environment schemes (AES) have been initiated in many countries [6]. AES aim to compensate farmers for the potential loss of income when they reduce intensity of production, with expected positive outcomes for biodiversity, ecosystem services and environmental pollution [7], [8]. AES measures cover a wide range of approaches, including in some regions the promotion of organic farming. Both organic farming, which explicitly forbids the use of chemical inputs, and other AES measures have been the focus of increasing numbers of studies in recent years, assessing their effectiveness in terms of biodiversity conservation, e.g. [7]. Many studies focus on the importance of AES for species richness and populations of endangered species, see e.g. the syntheses of [4], [5], [6], whereas relatively few deal with the effects on the functional composition of plant communities, such as pollination type, but see [9], [10], [11], [12]. Dependence on pollinators is an important plant trait facilitating genetic exchange [13]. Animal (mostly insect) pollination is an important ecosystem function, supporting 88% of all plant species across the globe [14]. However, disturbances such as intensive agricultural practices lead to disruptions of plant-pollinator interactions, and plant communities in disturbed environments are thus characterized by higher proportions of wind-pollinated species [15]. Gabriel and Tscharntke [16] showed that organic farming can benefit insect-pollinated arable weeds resulting in a shift in arable weed community structure towards a higher proportion of insect-pollinated species in organic crop fields. Similarly, Power et al. [17] found that insect-pollinated plants benefit more from organic management than non-insect pollinated plants in grasslands. Biesmeijer et al. [18] additionally showed that insect-pollinated plants and wild bees decrease in tandem.

In this study, we compared the species richness and cover of insect-pollinated vs. non-insect pollinated forbs in organic vs. conventional meadows and wheat fields along a landscape-scale management intensity gradient. To the best of our knowledge, there are no published studies on effects of organic vs. conventional management on insect vs. non-insect pollinated plant communities considering both arable fields and grasslands in their landscape context. Previous studies on individual agroecosystem types found a lower percentage of insect-pollinated vs. non-insect pollinated forb species in conventional than in organic management: for wheat fields this was ca. 9.0% lower [16] and for meadows this was ca. 5.5% lower. Thus we hypothesized that organic management favours insect-pollinated over non-insect pollinated forbs in wheat fields [16] and also in meadows [17], but with stronger effects in the more disturbed agroecosystem, i.e. in the wheat fields. In addition to effects of management type, we hypothesized that field edges support more insect-pollinated forbs than field centres due to influences such as lower management intensity and increased light availability [16]. Decreasing landscape-scale management intensity was also hypothesised to enhance insect-pollinated forb species and their abundance through increased propagule pressure from surrounding semi-natural habitats [19].

## Methods

### Ethics Statement

Permission to access fields and survey vegetation was obtained from all farmers.

### Study Area and Field Survey

We selected nine landscapes along a landscape-scale management intensity gradient (percent intensively used agricultural area, i.e. proportion of conventionally managed crop fields and grasslands, IAA: 48–98%) within a 35 km radius of the city of Göttingen, Lower Saxony, Germany (Fig. S1 in Supporting Information). In each landscape, a pair of conventional and organic winter wheat fields and a pair of conventional and organic permanent meadows were selected in close vicinity to each other (within-pair distance of wheat fields (mean ± SEM): 716±185 m; within-pair distance of meadows: 715±185 m; distance between wheat fields and meadows within the same landscape: 1101±109 m; distance between landscapes: 21.1±1.9 km). The two pairs per landscape resulted in 36 fields belonging to 24 farmers (most farmers managed mixed arable and livestock farms).

The study area is characterised by an agricultural mosaic of mostly intensively used arable crops and fertilised meadows, which also contains forest remnants and small fragments of semi-natural habitats such as calcareous grassland, naturally developed fallows, field margin strips and hedges. Around each field, the surrounding landscape was characterized based on official digital thematic maps (ATKIS DTK 50, year 2003) within a circle of 500 m radius using ArcGIS 9.2 (ESRI 2006). Prior calculating the landscape composition for each field, the thematic maps were improved based on field surveys. This distance has previously been found suitable to analyse landscape effects on plants in cereal cropping systems [20], [21]. The centre of the 500 m radius buffer was in the mid-point of the rectangle formed by the two transects in each field (see designation of transects below). There were in some cases overlaps of buffers within landscapes, but none between landscapes (Fig. S1). The proportion of intensively used agricultural area (% IAA) in a 500 m radius area did not differ significantly between organic and conventional fields for wheat fields or for meadows (t-test for paired samples, p>0.15).

The selected organic and conventional wheat fields received twice as much nitrogen fertiliser as meadows, while conventionally managed fields of both agroecosystems (i.e. meadows and wheat fields) received about four times as much fertiliser than organic ones (organic meadow (mean ± SEM): 29±19 kg N/ha; conventional meadow: 116±30 kg N/ha; organic wheat: 44±22 kg N/ha; conventional wheat: 209±22 kg N/ha). Organic fields were all managed without pesticides and synthetic fertilisers, and organic management had been practiced in all fields were older than 10 years. Conventional meadows were in a few cases treated with herbicides, and all meadows were improved meadows. Conventional meadows were mown nearly twice as frequently (2.8±0.3 times per year) as the organic ones (1.7±0.3 times per year), with the first cut in mid-May. 6 organic and 4 conventional meadows were additionally grazed in the fall (for more details on management and landscape structure see [22]). The yields for wheat fields were (mean ± SEM): organic wheat 44±5 dT/ha; conventional wheat 80±7 dT/ha.

In each field, one edge (in the first wheat row, or in case of meadows next to the edge) and one field interior transect (30 m into the centre and parallel to the edge in both agroecosystems) were surveyed in June 2008. In each transect four 5×1 m plots (288 plots in total) were established, spaced 12 m apart. Field edges were bordered by grassy field margins. Cover of each plant species (%), bare ground (%) and cover of cereal (%; only in wheat fields) was estimated in each plot. Subsequently, relative cover of each species and the total number of plant species (i.e. species richness per 20 m^2^) were recorded for each transect. Relative cover (%) per species was calculated by dividing the cover of the given species by total plant cover plus bare ground cover and also wheat cover in case of wheat fields. In the current study we exclusively focus on forbs, hence other plants (mainly grasses) were not included in the analyses.

### Statistical Analyses

In order to study the effect of a) landscape-scale management intensity (% IAA), b) management (organic vs. conventional), c) within-field position (edge vs. interior) and d) pollination type (insect vs. non-insect pollinated) on species richness and cover of forbs, we classified all forb species according to their pollination type in two groups: insect vs. non-insect (i.e. self and wind) pollination using the BiolFlor database [23]. Species were classified as insect-pollinated if the database stated that they can be also or exclusively pollinated by insects (many plants that normally are insect pollinated can also be e.g. wind-pollinated). Since forb cover was generally much higher in meadows than in wheat fields, we performed separate statistical analyses for these two agroecosystem types. Additionally, we further classified the forb species in bumblebee-pollinated vs. non-bumblebee pollinated forb species based on the BiolFlor database [23].

First, general linear mixed-effects models with Maximum Likelihood method for nested sampling were used to analyse the effects of the four explanatory variables (a–d) listed above and their two-way interactions on species richness and total relative cover of forbs. The following random factors were used (number of observations –72): landscape (9) and transect (36). Transect as random factor was included to account for the fact that the number of species and cover in each trait group were quantified at the same locations. The same analyses were performed for bumblebee-pollinated vs. non-bumblebee pollinated forb species. Calculations were performed using the nlme package (version 3.1 [24]) for R 2.11.1 [25]. Models were simplified with a stepwise model selection based on AIC by using ‘stepAIC’ function of MASS package [26].

Second, in order to test whether landscape-scale management intensity (% IAA), local management (organic vs. conventional) and within-field position (edge vs. interior) affect the community composition of forbs, we performed partial redundancy analyses (RDA). To characterise the community composition of forbs we used the relative percentage cover of each species. These analyses were done separately for insect-pollinated and non-insect pollinated forbs in order to investigate whether these factors affect pollination types differently. The species matrix was constrained by the predictor variables landscape-scale management intensity, local management or within-field position, while landscape (9 landscapes) was always included as a conditional variable (to account for nesting). Prior to the analyses, the species matrix was transformed with the Hellinger transformation [27]. This transformation allows the use of ordination methods such as PCA and RDA, which are Euclidean-based, with community composition data (site×species matrix) containing many zeros, i.e. characterised by long gradients. Pseudo-F values with the corresponding p values were calculated by permutation tests based on 999 permutations. Calculations were performed using the vegan package (version 2.0 [28]).

## Results

A total of 62 forb species were identified in the meadows, consisting of 40 insect-pollinated (38 in organic, 21 in conventional meadows) and 22 non-insect pollinated (21 in organic, 13 in conventional) species (Table S1). In the wheat fields altogether 57 forbs were identified, consisting of 33 insect-pollinated (29 in organic, 11 in conventional) and 24 non-insect pollinated (22 in organic, 11 in conventional) species (Table S2).

**Figure 1:**
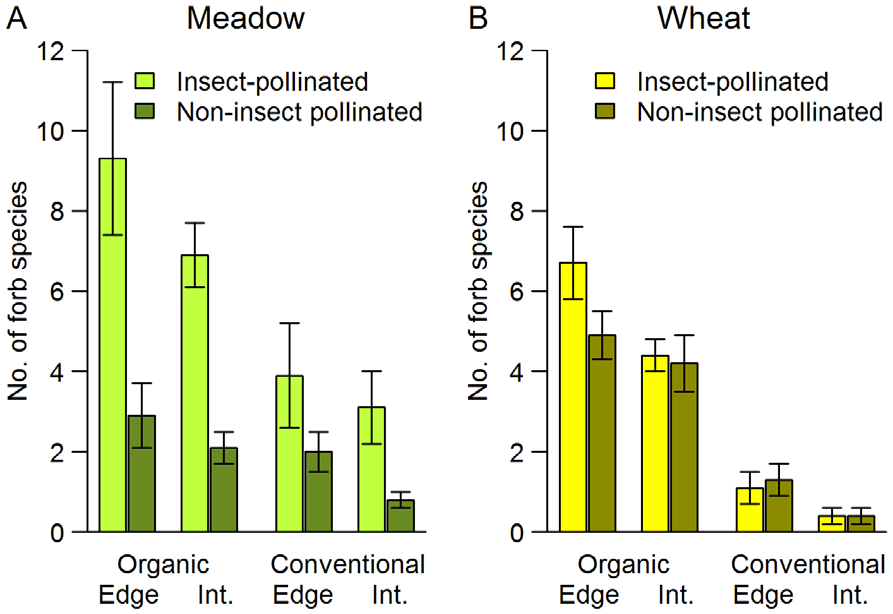
Mean (± SEM) forb richness in organic vs. conventional meadows (A) and in organic vs. conventional wheat fields (B). Data were gathered in edge and interior (Int.) transects of 20 m^2^.

We found a significant positive effect of organic management on species richness of forbs with higher richness in organic than in conventional fields (Table 1). This positive effect, however, was more pronounced for insect-pollinated than for non-insect pollinated forbs in both agroecosystems, resulting in an interaction between pollination type and management (Fig. 1a,b). Regarding this interaction, the relative change in the proportion of insect-pollinated vs. non-insect pollinated forb species was higher in wheat fields (28.1% decrease from organic to conventional management) than in the meadows (15.0%) (Table 1; Fig. 1a,b). Finally, species richness of forbs was higher in the edges than in the interiors in organic wheat fields for bumblebee-pollinated vs. non-bumblebee pollinated forb species revealed similar results, with the main exception that we found management×pollination interaction only in case of wheat fields (Table S3).

**Table 1:** Results of general linear mixed models testing the effects of landscape composition (intensive agricultural area %), agroecosystem type (meadow vs. wheat field), management (organic vs. conventional), position in field (edge vs. interior) and pollination (insect-pollinated vs. non-insect pollinated) on species richness and percentage cover of forbs in meadows and in wheat fields.

	Variable	df	F	p	effect
Meadow					
Species richness	Landscape	24	1.10	0.306	
	Management	24	13.95	0.001	C<O
	Position in field	24	0.23	0.143	
	Pollination	33	75.65	<0.001	IP> NP
	Landscape×Pollination	33	2.68	0.111	
	Management×Pollination	33	20.68	<0.001	
Cover	Landscape	23	0.41	0.526	
	Management	23	2.41	0.134	
	Position in field	23	0.32	0.577	
	Pollination	33	59.99	<0.001	IP> NP
	Management×Landscape	23	0.79	0.384	
	Landscape×Pollination	33	0.65	0.426	
	Management×Pollination	33	16.58	<0.001	
Wheat field					
Species richness	Landscape	24	3.98	0.057	
	Management	24	118.18	<0.001	C<O
	Position in field	24	7.92	0.010	E>I
	Pollination	33	0.04	0.845	
	Landscape×Pollination	33	1.23	0.275	
	Management×Pollination	33	4.20	0.048	
Cover	Management	25	102.78	<0.001	C<O
	Position in field	25	4.18	0.052	
	Pollination	34	5.46	0.465	
	Management×Pollination	34	1.99	0.168	

df: denominator degrees of freedom. Effect: direction of the significant effect (C: conventional, O: organic; E: edge, I: interior; IP: insect-pollinated, NP: non-insect pollinated).

**Figure 2:**
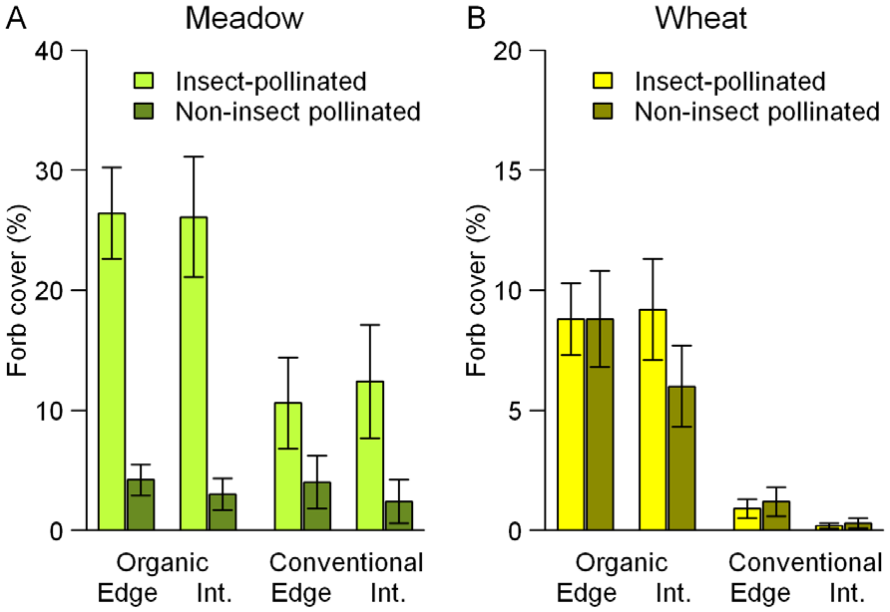
Mean (± SEM) forb cover (%) in organic vs. conventional meadows (A) and in organic vs. conventional wheat fields (B). Data were gathered in edge and interior (Int.) transects of 20 m^2^.

In wheat fields, the cover of both insect-pollinated and non-insect pollinated forbs was significantly higher in organic than in conventional management (Table 1; Fig. 2a,b). The interaction between pollination type and management on forb cover in meadows indicates that forb cover was enhanced by organic management predominantly in the case of insect-pollinated plants, but not so strongly for wind- or self-pollinated plants.

**Figure 3:**
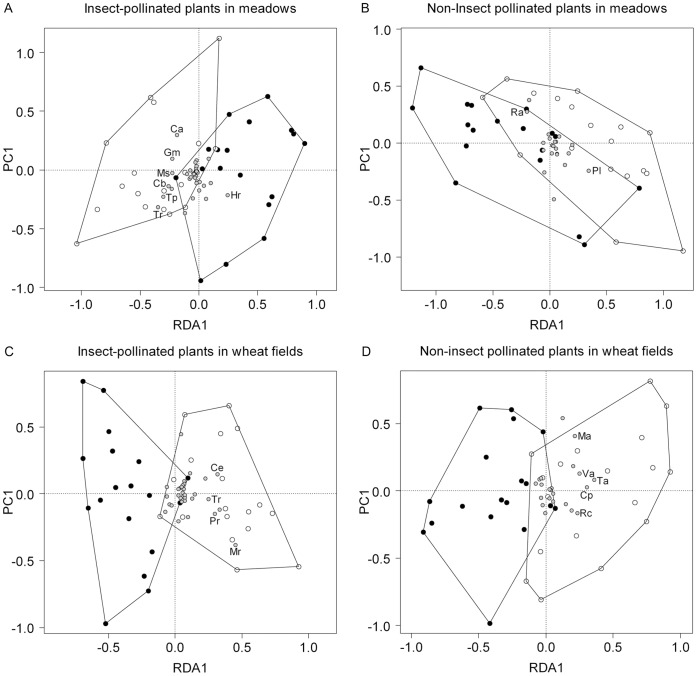
RDA plots for insect-pollinated and non-insect pollinated forbs in meadows (A, B) and wheat fields (C, D). White circles: plant survey transects in organic fields; black circles: plant survey transects in conventional fields; smaller grey circles: forb species with the highest fraction of variance (Ca: *Convolvulus arvensis*; Cb: *Crepis biennis*; Ce: *Cirsium arvense*; Cp: *Capsella bursa-pastoris*; Gm: *Galium mollugo*; Hr: *Hypochaeris radicata*; Ma: *Myosotis arvensis*; Mr: *Matricaria recutita*; Ms: *Medicago sativa*; Pl: *Plantago lanceolata*; Pr: *Papaver rhoeas*; Ra: *Rumex acetosa*; Rc: *Rumex crispus*; Ta: *Thlaspi arvense*; Tp: *Trifolium pratense*; Tr: *Trifolium repens*; Va: *Veronica arvensis*). Minimum convex polygons of the two management types are shown.

The partial RDA showed significant effects of management on the community structure of insect-pollinated forbs in both agro-ecosystems (Table 2; Fig. 3), altering the community composition almost completely. In organic meadows, the community composition was determined by a few characteristic species, such as *Crepis biennis* or *Trifolium spp.*, whereas *Hypochaeris radicata* was more common in conventional meadows (Fig. 3). In wheat fields, some characteristic species (e.g. *Cirsium arvense*, *Matricaria recutita*, *Papaver rhoeas*) occurred much more frequently in organically than in conventionally managed fields (Fig. 3). The community composition of non-insect pollinated forbs was also significantly affected by management, and in meadows transect position had also significant effect (Table 2; Fig. 3).

**Table 2:** Results of partial RDA to analyse effects of landscape (intensive agricultural area %), management (organic vs. conventional) and position in field (edge vs. interior) on species composition of insect and non-insect pollinated forbs in meadows and in wheat fields.

	Variable	Variation (%)	pseudo-F	p
Meadow				
Insect-pollinated forbs	Landscape	3.11	1.26	0.239
	Management	9.82	3.96	0.001
	Position in field	2.47	1.00	0.440
Non-insect pollinated forbs	Landscape	2.74	1.07	0.410
	Management	5.33	2.08	0.024
	Position in field	6.34	2.48	0.004
Wheat field				
Insect-pollinated forbs	Landscape	2.91	1.28	0.215
	Management	1.93	5.24	0.001
	Position in field	2.50	1.10	0.362
Non-insect pollinated forbs	Landscape	3.03	1.37	0.164
	Management	8.71	3.94	0.001
	Position in field	1.70	0.77	0.679

Percentage of explained variation, pseudo-F values and p values are given. Denominator degrees of freedom was 24 in all analyses.

## Discussion

Both diversity and cover of forbs were positively affected by organic management in meadows and wheat fields. This effect differed between pollination types for species richness in both agroecosystem types and for cover in meadows. We show for the first time in a comprehensive analysis (covering arable crops and grasslands paired in the same landscapes) that insect-pollinated plants benefit more from organic management than non-insect pollinated plants, regardless of agroecosystem type and landscape complexity. These benefits were more pronounced in wheat fields than in meadows. The positive organic management effect was also observed for non-insect pollinated plants in both agroecosystems. Additionally, we found that the community composition of insect-pollinated and non-insect-pollinated forb communities differed greatly based on the management type.

Both organic meadows and organic wheat fields contained disproportionally more insect-pollinated forb species than their paired conventional meadows and wheat fields. This supports recent studies showing a higher number of insect-pollinated plant species under organic compared to conventional management [9], [10], [16], [17]. The mechanism behind the difference in sensitivity to organic management could be that both pollinators and their food plants benefit from the absence of pesticide use, e.g. [7], [29], [30], [31], [32]. This supports the promotion of organic farming as a means to conserve farmland biodiversity and ecosystem services.

Comparing organic and conventional management, we observed a stronger decrease in the proportion of insect-pollinated vs. non-insect pollinated forb species in wheat fields than in meadows. In wheat fields, soil disturbance by annual ploughing or harrowing is likely to cause larger seed loss and larger extinction probability compared to permanent meadows [33]. In meadows, plant species once established can persist over long time periods also under conventional management. This can lead to less pronounced differences of species richness between the two pollination types in meadows. Finally, the lower light availability at ground level in wheat fields than meadows, may increase the sensitivity of forbs to the negative effects of increased fertilizer and pesticide input involved in conventional agriculture.

Recently, several studies have pointed to a link between declines of pollinators and insect-pollinated plants, e.g. [16], [34], [35]. Müller et al. [36] analysed the pollen requirements of European bee species and concluded that recent declines of bee populations are related to the decrease of flower diversity and quantity due to modern agriculture practices. Carvell et al. [37] also reported a national-scale decline in forage availability for bumblebees in the UK during the last century, but moreover showed that changes in abundance of forage plants were greater than those of non-forage plant species. This reflects both qualitative and quantitative decline of foraging resources of bees. These findings suggest that local management effects can cascade up to higher trophic levels including pollinators [22], [38]. Nevertheless, organic management can affect the plant-pollinator community from either direction: management enhances plant resources available for pollinators, while increased survival of the pollinator community benefits insect-pollinated plants. Our results support the findings that management-driven changes were stronger for insect-pollinated than non-insect pollinated forbs, which probably has consequences at much larger spatial scales. According to Holzschuh et al. [39], increasing landscape-wide percentage of organic fields results in higher flower resources and bee diversity even outside crop fields.

Furthermore, we found that community composition also differed between conventional and organic fields. Organically managed fields could be characterized by a few typical insect-pollinated species, such as *Trifolium pratense* in meadows and *Cirsium arvense* in wheat fields, which provide forage for bumblebees [37]. In contrast, in conventionally managed meadows or wheat fields just a mixture of accidentally occurring species was present. This was most likely because conventional fields were impoverished in both insect-pollinated and non-insect pollinated forb species, and therefore the likelihood of occurrence of common species is generally lower.

We did not detect effects of landscape structure on species richness and cover of forbs, which indicates that local management was a more important driver [10], [33]. Landscape-scale effects might be more important for mobile pollinators than for their less mobile food resources [39], [40], but see [19]. This is also reflected in a recent meta-analysis [5], showing that landscape strongly moderates the response of pollinators to management intensity.

At the smallest scale, i.e. within-field position, we found higher species richness of forbs of both pollination types in the edges than in the interiors. This is most probably because of the less efficient spraying of pesticides and fertilizers, higher light availability close to the borders [16] or mass-effects of higher propagule pressure from adjacent habitats.

In conclusion, our findings in both agroecosystem types (meadows and wheat fields) indicate that organic management supports high species richness and cover of insect-pollinated plants, which is likely to be favourable for the density and diversity of bees and other pollinators [41], [42]. These benefits were more pronounced in wheat fields than in meadows. Hence organic management contributes not only to biodiversity conservation but also increases resources for functionally important groups such as pollinators.

## Supporting Information

Figure S1 **Location of the sample fields.**
(PDF)

Table S1 **Overview of forb species, their pollination type and frequency in organic and conventional meadows.**
(PDF)

Table S2 **Overview of forb species, their pollination type and frequency in organic and conventional wheat fields.**
(PDF)

Table S3 **Results of general linear mixed models bumblebee-pollinated vs. non-bumblebee pollinated forb species analyses.**
(PDF)
