# Supplementary material for: Organic Farming Favours Insect-Pollinated over Non-Insect Pollinated Forbs in Meadows and Wheat Fields
Source: PLoS One. 2013 Jan 28;8(1):e54818. doi: 10.1371/journal.pone.0054818 (PMC3557276; doi:10.1371/journal.pone.0054818)
Supplement: Figure S1 — Location of the sample fields. (PDF) [file pone.0054818.s001.pdf]

Fig. S1. Location of the sample fields around the city Göttingen using ESRI World Imagery. Each dot represents one study site (green triangle: organic meadow, red triangle: conventional meadow, green circle: organic wheat field, red circle: conventional wheat field).

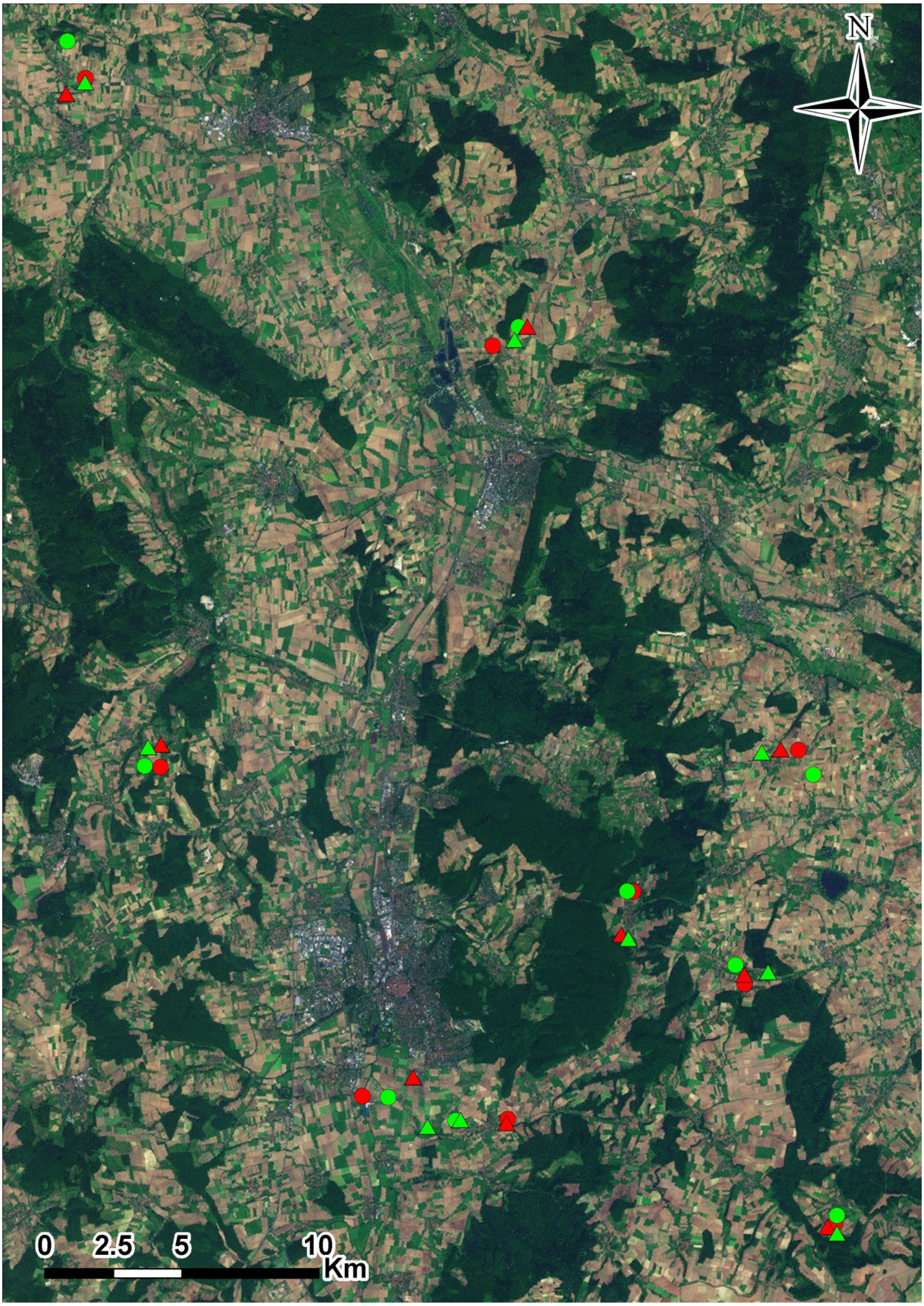

Upper left corner: UTM zone 32, Easting 550362 m, Northing 5745470.
Bottom right corner: UTM zone 32, Easting 584235 m, Northing 5697783.
