# Supplementary material for: Organic Farming Favours Insect-Pollinated over Non-Insect Pollinated Forbs in Meadows and Wheat Fields
Source: PLoS One. 2013 Jan 28;8(1):e54818. doi: 10.1371/journal.pone.0054818 (PMC3557276; doi:10.1371/journal.pone.0054818)
Supplement: Table S1 — Overview of forb species, their pollination type and frequency in organic and conventional meadows. (PDF) [file pone.0054818.s002.pdf]

Table S1. Overview of forb species, their pollination type (I = insect pollinated, N = non-insect pollinated) and frequency of occurrence in the edges and interiors of organic and conventional meadows. *: bumblebee forage plants.

Species	Pollination type	Organic		Conventional	
		Edge	Interior	Edge	Interior
Achillea millefolium*	I	3	1	1	0
Agrimonia eupathoria	N	1	0	0	0
Ajuga reptans*	I	0	2	0	0
Alchemilla vulgaris	?	0	1	0	0
Anthriscus sylvestris	I	2	0	0	1
Aphanes arvensis	N	1	0	0	0
Astragalus glycyphyllos*	I	1	0	0	0
Bellis perennis	N	0	1	0	1
Calystegia sepium	I	0	0	0	1
Capsella bursa-pastoris	N	0	1	0	1
Cerastium holosteoides	I	3	5	2	4
Cirsium acaule*	I	1	0	0	0
Cirsium arvense*	I	3	1	1	2
Cirsium vulgare*	I	2	2	0	0
Convolvulus arvensis*	I	6	5	1	2
Crepis biennis*	I	4	5	2	0
Daucus carota	I	2	0	0	0
Fragaria viridis	I	1	0	0	0
Galium aparine	N	0	0	2	0
Galium mollugo	I	4	2	0	0
Geranium dissectum	N	2	2	1	0
Geranium molle	N	1	0	0	0
Geum urbanum	N	2	0	2	0
Glechoma hederacea*	I	5	2	3	0
Heracleum sphondylium	I	0	1	1	1
Hieracium amplexicaule	?	1	0	0	0
Hypochaeris radicata*	I	0	1	4	2
Leontodon hispidus*	I	1	1	1	1
Leucanthemum vulgare*	I	1	0	0	0
Lotus corniculatus	I	2	0	0	0
Medicago lupulina	N	2	3	1	0
Medicago sativa	I	3	3	0	0
Myosotis arvensis	N	3	1	1	0
Pimpinella major	I	1	0	0	0
Plantago lanceolata	N	3	4	3	0
Plantago media	N	0	1	0	0
Polygala comosa	I	1	0	0	0
Polygonum aviculare	N	1	0	0	0
Potentilla anserina	I	2	0	1	0

Table S1. Continued.

Species	Pollination type	Organic		Conventional	
		Edge	Interior	Edge	Interior
Potentilla reptans	I	1	1	1	0
Ranunculus acris	I	4	2	2	3
Ranunculus bulbosus	I	0	1	0	0
Ranunculus repens	I	5	4	1	2
Rumex acetosa	N	0	2	1	3
Rumex crispus	N	3	2	1	1
Sanguisorba minor	N	1	0	0	0
Scabiosa columbaria*	I	1	0	0	0
Senecio jacobaea*	I	2	0	0	0
Stellaria media	N	0	1	0	0
Symphytum officinale*	I	0	0	1	1
Taraxacum officinale agg.*	I	9	9	7	6
Trifolium campestre	I	0	1	0	0
Trifolium dubium	N	1	0	0	0
Trifolium hybridum	I	1	0	0	0
Trifolium medium	I	1	0	0	0
Trifolium pratense*	I	5	6	2	1
Trifolium repens	I	4	6	2	1
Tussilago farfara*	I	1	0	0	0
Urtica dioica	N	3	0	4	0
Veronica arvensis	N	0	1	1	1
Veronica hederifolia	N	1	0	0	0
Veronica persica	N	1	0	1	0
Vicia cracca	I	1	1	1	0
Vicia sepium	I	1	0	1	0

?: not known.
