# Supplementary material for: Organic Farming Favours Insect-Pollinated over Non-Insect Pollinated Forbs in Meadows and Wheat Fields
Source: PLoS One. 2013 Jan 28;8(1):e54818. doi: 10.1371/journal.pone.0054818 (PMC3557276; doi:10.1371/journal.pone.0054818)
Supplement: Table S2 — Overview of forb species, their pollination type and frequency in organic and conventional wheat fields. (PDF) [file pone.0054818.s003.pdf]

Table S2. Overview of forb species, their pollination type (I = insect pollinated, N = non-insect pollinated) and frequency of occurrence in the edges and interiors of organic and conventional wheat fields. *: bumblebee forage plants.

Species	Pollination type	Organic		Conventional	
		Edge	Interior	Edge	Interior
Agrimonia eupathoria	N	0	0	1	0
Alliaria petiolata	N	1	0	0	0
Aphanes arvensis	N	2	4	1	0
Arabidopsis thaliana	N	1	0	0	0
Ballota nigra	I	1	0	0	0
Brassica napus	I	1	1	0	0
Calystegia sepium	I	1	0	0	0
Capsella bursa-pastoris	N	3	4	0	0
Centaurea segetum*	I	0	1	0	0
Cerastium holosteoides	I	2	1	0	0
Cirsium arvense*	I	8	5	2	0
Cirsium palustre*	I	0	0	1	0
Cirsium vulgare*	I	1	1	0	0
Conium maculatum	I	1	0	0	0
Convolvulus arvensis*	I	4	4	3	0
Euphorbia exigua	I	1	0	0	0
Euphorbia helioscopa	I	1	1	0	0
Galium aparine	N	5	4	3	1
Galium mollugo	I	2	0	0	0
Geranium pusillum	N	0	0	1	0
Geranium pyrenaicum*	I	0	0	1	0
Geranium rotundifolium	N	1	0	0	0
Geum urbanum	N	1	0	0	0
Glechoma hederacea*	I	1	2	0	1
Helianthus anuus*	I	1	0	0	0
Heracleum sphondylium	I	1	0	1	0
Lapsana communis	N	1	0	0	0
Lathyrus sativus	N	1	0	0	0
Matricaria recutita*	I	5	5	0	1
Myosotis arvensis	N	3	5	0	1
Papaver rhoeas	I	4	3	0	0
Polygonatum verticillatum	I	1	0	0	0
Polygonum aviculare	N	1	1	0	1
Polygonum persicaria	N	1	0	0	0
Potentilla reptans	I	2	0	0	0
Ranunculus acris	I	3	1	0	0
Ranunculus arvensis	I	0	0	0	1
Ranunculus repens	I	2	0	0	0
Raphanus raphanastrum	I	1	0	0	0
Rumex acetosa	N	3	1	1	0

Table S2. Continued.

Species	Pollination type	Organic		Conventional	
		Edge	Interior	Edge	Interior
Rumex crispus	N	3	3	0	0
Sinapis arvensis	I	1	1	0	0
Stellaria media	N	1	3	1	0
Taraxacum officinale agg.*	I	2	0	0	0
Thlaspi arvense	N	5	5	0	0
Trifolium pratense*	I	3	4	0	0
Trifolium repens	I	3	5	0	0
Urtica dioica	N	1	0	1	0
Veronica arvensis	N	4	4	0	0
Veronica hederifolia	N	1	1	1	0
Veronica persica	N	2	1	2	1
Vicia cracca	I	3	2	1	0
Vicia sativa	N	1	1	0	0
Vicia sepium	I	3	2	0	0
Vicia tetrasperma	N	2	1	0	0
Viola arvensis	I	1	1	1	0
Viola tricolor	I	0	0	0	1
