# Supplementary material for: Organic Farming Favours Insect-Pollinated over Non-Insect Pollinated Forbs in Meadows and Wheat Fields
Source: PLoS One. 2013 Jan 28;8(1):e54818. doi: 10.1371/journal.pone.0054818 (PMC3557276; doi:10.1371/journal.pone.0054818)
Supplement: Table S3 — Results of general linear mixed models bumblebee-pollinated vs. non-bumblebee pollinated forb species analyses. (PDF) [file pone.0054818.s004.pdf]

Table S3. Results of general linear mixed models testing the effects of landscape composition (intensive agricultural area %), agroecosystem type (meadow vs. wheat field), management (organic vs. conventional), position in field (edge vs. interior) and pollination (bumblebee-pollinated vs. non- bumblebee pollinated) on species richness and percentage cover of forbs in meadows and in wheat fields.

		Variable	df	F	p	effect
Meadow						
	Species richness	Landscape	24	0.49	0.489	
		Management	24	14.89	0.001	C < O
		Position in field	24	2.53	0.125	
		Pollination	34	12.24	0.001	Bb > NB
		Management × Pollination	34	1.27	0.268	
	Cover	Landscape	23	0.55	0.465	
		Management	23	18.09	<0.001	C < O
		Position in field	23	0.04	0.835	
		Pollination	34	1.59	0.216	
		Landscape × Pollination	23	2.39	0.136	
		Management × Pollination	34	1.92	0.174	
Wheat field						
	Species richness	Landscape	24	2.51	0.126	
		Management	24	121.28	<0.001	C < O
		Position in field	24	6.84	0.015	E > I
		Pollination	32	38.09	<0.001	Bb > NB
		Landscape × Pollination	32	0.07	0.794	
		Management × Pollination	32	59.94	<0.001	
		Position in field × Pollination	32	3.30	0.079	
	Cover	Landscape	24	3.63	0.069	
		Management	24	76.42	<0.001	C < O
		Position in field	24	3.91	0.060	
		Pollination	33	23.08	<0.001	Bb > NB
		Management × Pollination	33	24.14	<0.001	
		Position in field × Pollination	33	0.67	0.418	

df: denominator degrees of freedom. Effect: direction of the significant effect (C: conventional, O: organic; E: edge, I: interior; Bb: bumblebee-pollinated, NB: non-bumblebee pollinated).
